# A quantitative comparison of virtual and physical experimental paradigms for the investigation of pedestrian responses in hostile emergencies

**DOI:** 10.1038/s41598-024-55253-9

**Published:** 2024-03-22

**Authors:** Alastair Shipman, Arnab Majumdar, Zhenan Feng, Ruggiero Lovreglio

**Affiliations:** 1 Civil and Environmental Engineering, Imperial College London, London, UK; 2 School of the Built Environment, Massey University, Palmerston North, New Zealand

**Keywords:** Pedestrian dynamics, Emergencies, Experiments, Civil engineering, Engineering

## Abstract

Modern experiments investigating human behaviour in emergencies are often implemented in virtual reality (VR), due to the increased experimental control and improved ethical viability over physical reality (PR). However, there remain questions regarding the validity of the results obtained from these environments, and no full validation of VR experiments has yet appeared. This study compares the results of two sets of experiments (in VR and PR paradigms) investigating behavioural responses to knife-based hostile aggressors. This study quantitatively analyses these results to ascertain whether the different paradigms generate different responses, thereby assessing the use of virtual reality as a data generating paradigm for emergencies. The results show that participants reported almost identical psychological responses. This study goes on to identify minimal differences in movement responses across a range of predictors, noting a difference in responses between genders. As a result, this study concludes that VR can produce similarly valid data as physical experiments when investigating human behaviour in hostile emergencies, and that it is therefore possible to conduct realistic experimentation through VR environments while retaining confidence in the resulting data. This has major implications for the future of this type of research, and furthermore suggests that VR experimentation should be performed for both existing and new critical infrastructure to understand human responses in hostile scenarios.

## Introduction

There a numerous examples of emergency situations where an informed model of human movement responses would be beneficial, for example in understanding evacuations from natural disasters, terrorist attacks, or crowd crushes^1^. However, while there have been many attempts, a fully validated, predictive model does not yet exist^2^.

Pedestrian dynamics approaches try to characterise and model human movement in arbitrary scenarios^3^, with common use cases being fire evacuation^4^, level-of-service estimation^5^, and large-scale event modelling^6^. However, these models depend strongly on calibration data to predict likely outcomes^7^. This data can come from real-world scenarios, which is sparse, uncontrolled and often sensitive, or from experimental approaches, which is difficult to generate, ethically difficult, and scenario specific^8,9^. Experimental approaches for emergency scenarios have been developing over the past decades, with many previous examples of data generation, including physical reality (PR)-based experiments (such as drills, laboratory experiments, and animal experiments), and virtual reality (VR)-based experiments (ranging from non immersive desktop-surveys to fully immersive environments)^10,11^.

While there is a lack of definitive data on how crowds respond, it is strongly suggested that different emergencies will produce different reactions. For example, it is unknown^12^ whether a fire might result in very different movement responses to those seen in during a knife-based attack, which in turn are different to those produced by a bombing or a Marauding Terrorist Firearm Attack (MTFA). This study only examines the responses to hostile, knife-based attacks, and the development of the methodology to investigate these attacks has been detailed further^13^.

There are many considerations for performing PR experiments^14,15^. Important considerations include: time and space constraints on any procedures, ethical limitations (e.g. difficulties in investigating human responses to explosions), and further uncontrolled confounding variables. Conversely, VR offers the opportunity for completely controlled and repeatable experiments, allowing the investigation of extremely dangerous scenarios (where participants would be at physical risk, such as a building fire^16^) within a safe environment, potentially with a smaller logistical requirement^17^. The increasing quality of VR hardware allows the development of near-identical environments to real-world scenarios, which can be repeated perfectly for different participants^18,19^. There are logistical requirements in performing these experiments, including the time taken to develop the environment and the financial outlay for the equipment. However, this offsets the requirements for physical experiments, where experiment times are usually inflexible, and experiment venues can be expensive to hire or be difficult to access^20^.

While VR therefore represents a great opportunity, there are limitations in performing experiments within VR^21^, including in measuring the interactions between participants^22^. A primary, inherent issue is the fact that participant actions and choices are limited to how the VR environment is implemented. There is, consequently, less flexibility for the participant’s action choice in VR in comparison to physical experiments. For example, crypsis, or ‘playing dead’, is a possible approach for participants in a PR experiment, but becomes a particularly complex problem for implementation within a VR environment^13^. Another major, although not inherent, issue is that there are concerns regarding the validity of any data produced within VR environments, when compared with PR environments^23–25^. As such, there is no consensus over the most appropriate manner of investigating these events, and few comparisons have been made between paradigms.

There is little data surrounding the validity of VR as a data generating paradigm^26^. This has limited the potential research and progress, as VR is far more ethically viable than PR, owing to the much reduced physical risk (while admittedly introducing potential motion sickness risks). There are numerous further advantages of VR, including the potentially reduced logistical requirements, repeatability of experiments, and limits in measurement noise. Therefore this study aimed to compare data generated from both paradigms, performing a validation exercise on VR as a data-generating paradigm. This study performed two sets of experiments, one VR and one PR, for the same emergency scenario. These experiments investigated pedestrian movement responses to knife-based hostile attacks, and were designed to be as identical as possible, including identical stressors and obtained measures. These results of these experiments are then compared, identifying any differences in participant psychological and movement responses.

The data compared in this paper was produced by two separate experimental procedures. The generation of the VR dataset is discussed here. The comparison of these two datasets is one of the first ever quantitative comparison between near-identical experimental procedures, providing a useful insight into the utility of VR approaches for investigating human behaviour in emergencies.

This paper will initially produce a literature review, before continuing to detail the different experiments performed and the proposed measurements and analysis methods. It will then highlight the differences between the two paradigms and then present the results from the two experimental processes, before finally discussing the implications of these results.

## Literature review

Historical attempts at investigating responses to stressful stimuli have varied from laboratory experiments with the threat of electrical shocks^27,28^, financial incentives^29^, animal experiments^30^, hypothetical choice experiments^31^, and narrative-based approaches^32^. Expanding on these, there have been many PR-based studies with human participants in controlled environments that investigate emergency movement responses, such investigating financially-incentivised movement through a plane evacuation^33^, or a fire evacuation from a hotel^34^. One notable experimental example to investigate terrorist attacks is seen in the work performed by Li et al.^35^, who investigated the initial movement responses from individual as a result of the placement of a knife-wielding actor in randomly selected locations surrounding the participants. While this is an example of the high potential of physical experiments, this study was limited in many ways. These limitations include the fact that participants were fully informed (as most terrorist attacks are unannounced), a lack of any emotional measures to validate the responses, and the use of a single participant at any a time, precluding social interactions in the responses.

VR has been used for decades in multiple formats, varying from basic computer screen simulations (for example, Second Life^36^ and computer games) to highly immersive virtual environments (IVEs). VR is also becoming an emerging tool to investigate human behaviour in emergencies using both immersive and non-immersive solutions^37,38^. However, there remains several unanswered research questions surrounding the use of VR as a data-gathering tool, especially when considering behaviour in emergencies. Several studies have provided partial answers on this subject as well as provide insights into the ecological validity of data generated using VR experiments. This section provides an overview of the most relevant studies.

VR has been used extensively in the past to investigate human responses to emergency scenarios. Moussaid et al.^39^ provide varying financial incentives to move through a bottleneck within a corridor, measuring herding and other social effects while varying levels of ‘stress’. Another serious games approach by van den Berg^40^ allowed participants to choose a type of transport after hearing an in-game alarm. This study also allowed participants to react to choices made by other participants, measuring a herding effect. Lin et al.^41^ also produced a study looking at how participants in an IVE responded to avatar movement during an emergency in a railway station.

One major requirement when considering the validation of VR is the social element of any behavioural responses, where participants’ choices depend on their observations of their neighbours. A common source of contention surrounding the results of VR experimentation is the fact that participants often know that their neighours are in fact computer controlled, resulting in less of a social contract. There are numerous examples of VR experiments obtaining results where participants followed social cues from agents they knew to be computer-controlled. An example approach by Song and Lovreglio^42^ investigated discrete choice behaviour of building occupants during a fire evacuation, utilising a virtual reality experimental paradigm and varying the density of avatars at different exits. A further example by Lin et al.^43^ showed how individual movement in a train station evacuation can be affected by observed movement of non-player characters (NPCs). Crucially, these examples all suffer from a lack of quantitative real-world data to compare against, which presents a difficulty when performing the validation of VR as a data-generating paradigm.

Previous research has attempted to use the concept of presence as proof that VR-generated data is valid for use in real-world contexts, with a high level of presence implying that any behaviour and responses mimic those that would occur in reality. However, this approach has limitations, including a difficulty in quantitative measurement and little hard proof of the relationship between presence and validity.

Recently there have been several VR-based experiments investigating the movement of pedestrians during emergencies, such as^16^. A relevant recent study was performed by Awada et al.^43^, focusing on using heart rate and self-assessed questionnaires to assess emotional responses in VR, while participants moved in response to an active shooter. This study investigated different locomotion methods, identifying that walking in place was the method that allowed the highest sense of presence and produced the largest emotional responses.

A 2014 study by Kinateder et al.^21^ performed a strengths, weaknesses, opportunities and threats (SWOT) analysis examining these, concluding that while there are large potential benefits to using VR as an experimental paradigm, further validation should be performed before any results can be incorporated into the existing body of research. Subsequently, Feng et al.^44^ performed a systematic review examining how VR has been used, focusing on emergency evacuations and pedagogical research. They concluded that there has been a prior focus on using VR for fire-related emergency evacuations, and that therefore there is an opportunity for the use of VR to investigate further emergencies, such as terrorist attacks. A more recent review by Feng et al.^38^ raises questions on how whether participants’ behaviour in VR environments is consistent with their behaviour during real-life emergencies (i.e., PR).

The existing literature shows an increasing trend of VR studies used to investigate how humans behave and make decisions in emergencies^37^. However, only a few studies investigate the validity of immersive and non-immersive VR behaviours and choices by comparing them against real-world data collected in the PR. One of the first attempts was carried out by Kobes et al.^45^ investigated the impact of smoke on exit choice in both VR and field-based hotel evacuations. This experiment used a non-immersive setup, and the results show that in some scenarios, the choices made in VR were consistent with PR, while in other scenarios, the authors observed different behaviours. Similarly, Li et al.^46^ used a non-immersive VR setup to compare the data generated in the virtual environment with data collected in PR when investigating route choices. This study shows a qualitative agreement in results between the field study and the virtual experiment. In more recent years, new studies compared data from PR with the data generated using more immersive VR setups. For example, the study performed by Feng et al.^47^ used an HMD with $360^{\circ }$ videos to investigate exit choice behaviour in VR before comparing these responses to field experiments. This study also found quantitatively similar responses between the two experimental conditions while noting that further validation is required. Another study comparing wayfinding and pre-evacuation data from two immersive VR experiments and PR is the one carried out by Arias et al.^48^. In this study, the authors collected VR data using both cave automatic virtual environment (CAVE) and one using a head-mounted display (HMD). The results show agreement between PR data and HMD in terms of pre-evacuation time and exit choice. On the other hand, the authors observed a large difference between the PR data and the results from the CAVE experiment.

While most of the existing studies have been mainly focusing on wayfinding, there have been studies investigating the validation of VR responses against real-world data, mainly focusing on pre-evacuation behaviours. One of the first studies is the work by Kinateder and Warren^49^, who investigate the impact of social influences on the decision to start evacuating. Their results show an agreement between the VR and PR data supporting the ecological validity of VR as a research tool to study evacuation behaviour. Arias performed seminal work in understanding the potential for behavioural realism in VR environments across fire evacuation contexts^50^. Arias et al.^51^ investigate the pre-evacuation actions in a VR experiment based on the conditions of in a hotel room during the MGM Grand fire in 1980. The VR data is compared with the data from one of the survivors of the real fire showing that the observed actions were similar, but there was a difference in terms of frequency at which those actions were performed. Finally, research performed by Feng et al.^47^ used an HMD with $360^{\circ }$ videos to investigate exit choice behaviour in both VR, before comparing these responses to field experiments. This study found quantitatively similar responses between the two, while noting that further validation is required.

A separate approach involves the comparison between VR and predicted real-world behaviour. One such example was performed in 2013 by Slater et al.^52^, who examined the use of IVE to examine bystander responses to violent emergencies. In this study, participants would observe virtual avatars engaging in violent confrontations, and the participant actions would be recorded and compared against the hypothetical actions predicted by the bystander effect.

The existing literature shows the potential of how VR can be a valid data-gathering tool for behavioural studies in emergency conditions. However, most of the existing case studies have been focused mainly on fire emergencies or the investigation of wayfinding decision-making. As such, the literature shows a lack of validation studies for hostile emergencies. Although there are already several VR applications looking at these types of emergencies to either investigate human behaviour or train people^53,54^, these existing works do not provide a comparison between VR and PR data. As such, this study will bridge this research gap providing by providing new insights into if and how VR is a suitable tool to investigate hostile emergencies. Finally, in line with the previous studies, two hypotheses are made here:

1. H1: In hostile emergencies, the participants’ emotional responses observed in the physical reality (PR) and virtual reality (VR) settings are equivalent.
2. H2: Significant differences exist between certain factors affecting behavioural responses from both PR and VR settings regarding the intensity of the reactions they elicit. In other words, the intensity of responses provoked by the factors in both settings can differ significantly.

To conclude this review, VR represents a huge opportunity for investigating how humans behave in emergencies, and therefore for advancing research in this area. However, it is relatively poorly understood how participants will act within VR when comparing against PR-based experiments, and also when comparing against the reactions of a participant to NPCs versus human neighbours. This lack of understanding has limited the research in this field, preventing the widespread use of VR-generated data in real-world contexts. This study was designed to quantitatively compare the responses of individuals in PR and VR experiments, providing some certainty over the usability of such data.

## Methods

This section will describe the two separate paradigms, initially focusing on the PR experimental procedure (Study 1) before moving on to the VR protocol (Study 2). The full PR protocol, including the design of the stressors and the experimental branches, is provided in^13^. The description of the VR experimental protocol includes the environmental design, the locomotion method, and the considerations behind the participant experience. This section then continues to summarise the participant pool and the measurements obtained including a power analysis and the comparison methodology to compare between the two paradigms. Finally this section describes any unavoidable differences between the two experimental procedures.

### Study 1: Physical reality experiment design

**Figure 1:**
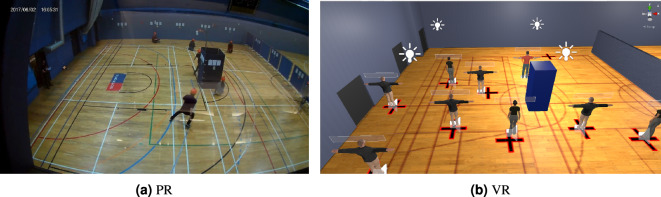
PR and VR environments.

The PR experiments were performed over the period Monday 17th to Friday 21st December 2018, over ten separate sessions. These sessions were carried out with participants in groups, ranging in size from 5 to 11 participants (mean 8, SD 1.789). In total 80 participants (26F, 54M) took part in these experiments. The experimental protocol was developed using a pilot study to approximate the conditions of a knife-based terrorist attack, while remaining within ethical boundaries. The final design required naïve participants, who were promised the ability to earn up to £40 as a result of the experiment (their financial incentive). They were told that if they failed the experiment, they would only be paid £5 (the financial stressor), without being told what conditions would lead to failure. Finally, after 5 min of distractor tasks, a hostile actor was introduced to the environment, loudly and aggressively explaining that if the actor managed to touch the participants then they would lose their financial incentive, before attempting to touch the participants. The participants’ movement reactions as a result of the introduction of this hostile aggressor were recorded, as well as their psychological responses. These results were then compared against the results of several control experiments, concluding that the participants had been stressed specifically by the introduction of the hostile aggressor. Finally, within the experimental groups there was a further intervention, where an actor within the group either attempted to evade the aggressor, or remained still. This difference provided the basis for a logistical model to understand the factors influencing the decision to move, or the ‘Flight-Freeze’ response. An example snapshot of the PR experiment environment can be seen in Fig. 1a, alongside a snapshot of the VR experiment from an equivalent angle in Fig. 1b. For a generalised approach for investigating terrorist attacks, and a full description of this experiment and resulting datasets, the reader is directed to^13^.

### Study 2: Virtual reality experiment design

The set of virtual (VR) experiments were performed between 14th January and 24th February 2020. In total this study performed 55 separate VR experiments, with the participants recruited from Imperial College London student and staff populations. It took approximately 6 months to design this experiment and obtain permission from the Imperial College Research Ethics Committee (ICREC reference: 19IC5216). This experiment was considered lower risk than the PR experiment, owing to the lack of movement and the virtual nature of the experiment.

Each experiment lasted approximately 90 min, including the set-up and break-down of experimental equipment, and required the presence of a single investigator to perform. This experiment was performed using an HTC Vive Pro Eye, in a room that measured approximately 5 m $\times$ 5m. The VR equipment was supported by an Alienware Aurora R8 desktop computer, with an Intel Core i9 9900 K CPU, and a Nvidia GTX 2080Ti GPU. The environment was built in Unity version 2019.1.10f1.

#### Environment

The virtual environment used in this experiment was designed to mimic that of the PR experiment as far as practicable. To this end, the blueprints of the sports hall building used within the PR experiment were used to create the virtual environment, leading to an almost identical setting. The virtual environment was created from a CAD file, before being transformed into a 3D model using Revit. The materials and lighting were modelled directly in Unity. This methodology was similar to that used in previous studies^55,56^.

#### Locomotion technique

The ability for the user to move in VR has a significant impact on the functionality and validity of the environment. The navigation methods cause the movement of the user’s avatar in the virtual environment, consequently updating the display shown to the user. However, unrealistic movement techniques can lead to increased motion sickness. Continuous movement responses with variable speed are required for the high resolution data and analysis used for pedestrian dynamics models. To achieve this, the locomotion technique used in this experiment was based on arm movement, provided by the open-source Virtual Reality Tool Kit (VRTK)^57^. In order to move, the participants held down the trigger button on the controller, and move their arms as though they were walking. The speed of movement in the environment was dependent on the speed of the arm movement, and the direction of movement was defined by the average direction of the controllers. This locomotion technique allowed the participants to move at variable speed and in any direction, while being decoupled from their gaze direction. This allowed the participants to move through the environment by swinging their arms, in a similar manner to walking and running. Further movement could be achieved by the participant physically walking around the environment. The investigator was present at all times to ensure that the participant was not at risk of harm by walking into walls or tripping over.

#### Non-player characters

**Figure 2:**
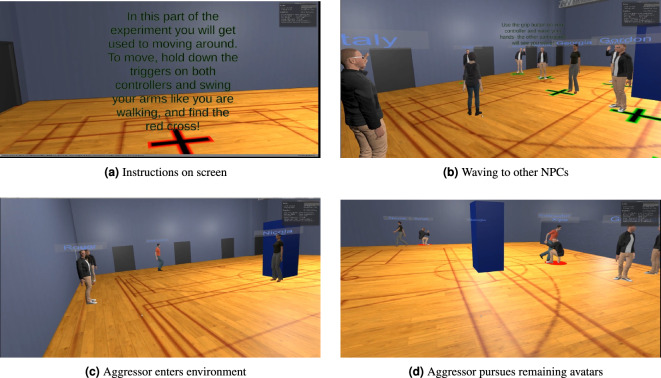
VR experiment snapshots.

The non-player character (NPC) avatars were produced using the online tool Adobe Fuse, with animations such as walking, running, strafing, and waving produced from the similarly online open-source tool Mixamo. Four separate NPC designs were used in this experiment, comprised of two female avatars and two male avatars. The NPC participants used two of these (one male and one female), the aggressor was a male avatar, and finally the instructor was a female avatar. Examples of these avatars are shown in Fig. 2.

To ensure a valid environment, the movement of NPCs was determined by trajectory data obtained from the PR experiment. During the experimental phase the NPCs followed pre-defined paths, while if at any point they collided with the participant they replotted their paths, speeding up and manoeuvring to rejoin their initial trajectory.

#### Social responses

There are well established differences surrounding the change between online and in-person interactions (for example, a conversation in person and a conversation over the internet can take drastically different courses^58^). Therefore, when considering investigating social effects in VR with the intent of using this data with real-world and physically interactive applications, investigators should control how participants view their neighbour agents. Indeed participants should ideally view these agents as other human participants with whom they will have a continued interaction.

There are several possible ways to achieve this, each with different requirements and associated levels of complexity. The ideal method is to implement a multiplayer VR environment, ideally with the participants co-located in the same experimental venue (rather than, say, connected across large distances by an internet connection), to ensure an ongoing social element to the experiment. This must be considered against the increases in environment complexity, logistical requirements, and financial cost.

A second method is a reverse Wizard-of-Oz study, implementing sophisticated NPCs while informing participants that they are human-controlled. This is a very complex task, requiring the NPCs to pass a form of Turing test^59^. Modern AI engines are not yet of high enough quality to fully implement a convincing NPC, and the task becomes more complex with every added element of functionality. This can be mitigated by limiting the possible interaction between participant and NPCs, with specific elements designed to increase the participants’ level of belief in the NPCs being controlled by other human participants. For example, a simple waving animation can be implemented, or a pre-recorded audio file played. The participants’ level of belief can be increased by implementing ‘mistakes’ in the interaction, for instance multiple waving animations, or a delay before the playing of the audio file. This method was used in this experiment.

The VR experiment was designed to mimic the PR experiment as far as practicable. However, an obvious difference to participants was that they were not taking part as a group, but rather as an individual in a room. This would usually lead the participant to believe that they were the only human participant in the experiment, which could potentially lead to different behaviour than that expected of a participant who is part of a set of human participants. Therefore over the experimental process several performative elements were implemented to convince the participant that the NPCs within their environment were in fact controlled by other human participants. The participants were all:

1. Informed that the other avatars in the environment were controlled by other participants, who were in other rooms in the same building;
2. Informed that they would be meeting the other participants after the experiment;
3. Asked to type their names into the computer, after being informed that it would be displayed above their avatar (as seen in Fig. 2). They were told that this was so that they could identify the other avatars, and that they would in turn be identifiable;
4. Asked to record an audio introduction, detailing their name, their subject, and in which room they were currently located.

During the experimental introduction section, all participants were:

5. Invited to play their own introduction audio file. The NPC audio files were all unique and each experiment provided the same avatar introductions.
6. Invited to ‘wave’ to the other NPCs. At a specific point in the experiment the NPC avatars were animated as waving to each other several times (ranging from one to three waves, separated by a fixed duration ranging from 0.2 to 1.0 s). As the NPC waves were predetermined, each experiment was identical in this respect.

The level of participant belief in the humanity of the observed avatars was measured after each experiment, after the participants had completed the final pieces of data collection and before they were informed of the artificial nature of the NPCs, as detailed in “Results” section.

#### Study procedure

The VR experiment recruited 55 participants (37 male, 18 female) using the same recruitment method as the PR experiment. There was a financial incentive provided in this experiment of £40 (identical to that the PR experiment). Participants were informed that if they successfully completed the experiment they would be paid the full amount, but if they failed for any reason, they would only be paid £5. This financial stressor aimed to produce a replicate a sense of urgency in the experiment, as detailed in^13^. All participants were paid the full amount, regardless of performance in the experiment. Prior to the experiment, each participant completed an identical questionnaire, including demographics and personality measures.

The VR experiment utilised movement data obtained from two separate PR experiments, and the hostile aggressor and NPCs followed routes defined by this data. In the first branch, participants (n = 27) observed the NPCs responding to the hostile aggressor and trying to flee. In the second branch, participants (n = 28) instead did not move at all in response to the hostile aggressor. The experiments were chosen as they represented the two extremes of the observations from the PR experiments.

The VR experiment was performed with one participant at a time, with each individual experiment taking approximately 90 min. The experiments were split into three phases, the pre-experiment phase, the experiment phase and the debrief phase. During the pre-experiment phase, the participants were provided experimental paperwork, including an information sheet and consent form, before providing initial self-assessed survey responses. At this point the participants were asked to record the introduction that would be played during the experiment. This introduction lasted for 7 s and could be re-recorded if necessary. They were then placed into the experimental area, at which point they were randomly allocated to an experimental branch.

Once within the environment the participants performed some acclimatisation exercises in an introduction phase (see Fig. 2a). When the participant had completed these exercises the NPC ‘participants’ enter the virtual environment, followed by an investigator. The investigator moves to the centre of the environment, and is identified with a green ring around their feet, before asking participants to move to the red cross locations. At this point the NPCs are automatically routed to pre-defined red crosses that appear on the floor. Once the participant has also reached a red cross, the NPCs and participant are identified one by one as a green highlighted circle appears at their feet and are asked to introduce themselves by playing pre-recorded audio files. They introduce themselves in order of distance from the investigator, with the nearest avatar beginning. The NPCs introduce themselves after a fixed delay, ranging from 1 to 3 s. Once the participant is identified, a button appears on the participant’s screen, asking them to click a button to introduce themselves. After the participant and the NPCs had introduced themselves, they are informed that they can wave at each other. The participant is then presented with a prompt on their screen, asking them to click the grip button on their controllers, and wave their hands (Fig. 2b). The participant observes the NPCs waving at this point, with pre-defined delays and repetitions. After the investigator had finished giving out the instructions, the participant is presented with a prompt on their screen, asking them to find a red cross on the floor in order to start the experiment. Once the participant had found the red cross, they are given a distractor task of waving at the nearest avatar. After a fixed-time delay of 2 s an aggressor avatar entered (Fig. 2c) and loudly explained his role, saying.

HEY EVERONE. THAT’S RIGHT, FUCKING LISTEN UP. IF I TOUCH YOU, YOU WILL LOSE THE EXPERIMENT, AND LEAVE WITH NO MONEY. YOU UNDERSTAND? YOU CAME HERE WITH £40, AND IF I TOUCH YOU, YOU WILL LEAVE WITH £5.[Pointing at avatar in the opposite corner to the participant] RIGHT YOU, I’M GOING TO TOUCH YOU

The aggressor’s actions are identical to those from the PR experiment, including the initial target. At this point, the aggressor then pursues all NPCs and the participant, selecting the next target by proximity. If the aggressor moves into close contact (< 1 m) of the NPCs, they are prevented from moving further, placed into a crouch position, and highlighted in red (Fig. 2d). If the aggressor moves into close contact of the participant, then the participant is removed from the environment and presented with a prompt to take off the headset. The experiment was limited to 2 min, at which point it would end and the debrief process would take place.

During the debrief phase, the participants were asked to fill in post-experiment questionnaires. They were also provided with a further, post-experiment information sheet, detailing the true aims of the experiment and a re-consenting form.

### Measurements and proposed analysis techniques

This section details the different measurements obtained within the experimental procedures, as well as the analysis performed for each individual measure. These measures include psychological measures to establish cognitive states, and spatial measures to understand movement behaviour. This section then describes the methodology used to quantitatively compare the experimental paradigms.

#### Demographics and psychological measures

Standard participant demographic measurements were obtained prior to the experimental procedure. These included: age, exercise level, personality (measured by a ‘Big 5’ questionnaire^60^). The following significance codes were used: *p < 0.05, **p < 0.01, ***p < 0.001, and ^†^p < 0.1. Where the data was normally distributed, the demographic and psychological measures were compared with Welch’s t-test. Where there were deviations from normality, the Mann-Whitney test (indicated by the prefix ‘MW’) was used for comparisons. Further explanations and details of these assessments is provided in^13^.

A personality questionnaire was required to assess any differences in participant pool due to the impact of personality on participant risk perception which is one of the key factors affecting people’s response in emergencies according to the well-established Protective Action Decision Model^61,62^. In line with this conceptual model, several existing studies have shown that personality traits can make a significant contribution to evacuation choices in different types of emergencies^63^. As such, the ‘Big 5’ questionnaire was used in this research to assess if the participants of the PR and VR experiments have similar personality scores. The emotional questionnaires (STAI-T, STAI-S, and PANAS) were selected due to their extensive history and validation studies. Also in this case, we measure as previous research highlighted that emotional states, such as anxiety, can be a predictor of risk perception and evacuation response^64^.

This specific questionnaire was selected due to the large body of validation and repetition studies, as well as the quantitative nature of the personality scores. The exercise level was measured on a 1–5 Likert scale (1 = Not at all, 3 = once a week, 5 = more than three times a week). An exercise score was included to assess whether an individual’s level of exercise would change how they responded given the physical differences between a PR and VR experiment. Two well-established surveys were used to assess psychological responses to the experiment: a pre-experiment cognitive state, measured by the short form State Trait Anxiety Inventory- Trait (STAI-T)^65^, a post-experiment short form STAI-State (STAI-S) and the Positive and Negative Affect Schedule (PANAS)^66^. The STAI surveys assesses negative emotional states (anxiety) while the PANAS produces a measure of both positive and negative emotional states. The emotional questionnaires (STAI-T, STAI-S, and PANAS) were selected due to their extensive history and validation studies. To test the equivalence hypothesis (see H1 in the “Literature review”), we use the two one-sided tests (TOST) procedure. This equivalence test is used to statistically reject the presence of effects which is large enough to be considered worthwhile^78^. The following significance codes were used to report the results: *p < 0.05, **p < 0.01, ***p < 0.001, and ^†^p < 0.1.

#### Movement responses: discrete and continuous

Analysis of the participants’ movement required position measurements throughout the experiment. Participant position in the PR experiment was sampled using the Pozyx ultra-wideband (UWB) system, with a UWB tag providing measures of position (accurate to $\pm \, 0.1$ m) and rotation ($\pm \, 0.5^{\circ }$) at a 10 Hz update rate^67^. Participant position and orientation in the VR experiment was sampled directly from the environment, at the same 10 Hz frequency.

Two separate modelling approaches were pursued with these datasets: a discrete approach and a continuous approach. The discrete approach modelled the decision of individual participants to move or remain still (analagous to the ‘Flight-Freeze’ response), using a logistic model. The logistic modelling solution was selected as it is one of the most valuable modelling tools to investigate and predict discrete choices (e.g., binary of choices) in many fields such as transportation and human behaviour in emergencies and evacuation^68,69^. The data used to fit the discrete models was collected at snapshots after the aggressor entered the room whenever a participant moved for the first time and whenever the aggressor removed a participant from the experiment. For more information on this data collection procedure, refer to^13^. The model itself predicts the probability that a participant will move, or remain still, at each measured time point. The independent variables used to inform this model were selected due to their suggested importance when considering an individual’s response to a threat. These variables assess the impact of social influence^70,71^ on a participant’s discrete choice by measuring the number of participants already moving (‘Number reacted’), and the number already caught by the aggressor (‘Number caught’). The selected variables also account for the relative location of the aggressor by using relative distance (‘Distance to aggressor’) and whether the aggressor was currently moving towards them (‘Within FOV’-defined by within a $45^{\circ }$ field of view). Finally, the demographic of the participant was used, including the gender, the exercise level, and the age, as previous studies have highlighted how the characteristics of the decision-makers can have significant impact in their risk perception and response^72^.

The continuous approach modelled the movement of all individuals who had begun to move via a multivariable regression model. The data used to fit the continuous models was re-centered and rotated to the perspective of the aggressor. To further understand this, multivariable linear regression models were used to understand these responses from a total and radial perspectives. To build these models, both PR and VR datasets are standardised to zero mean and unit variance before combining into a single dataset for regression. The continuous models themselves quantitatively describe the observed motion with the relative position of the participant to the aggressor, accounting for direction, assessing all possible combinations of symmetric and asymmetric measures. In other words, they try to explain the participants’ motion as a response to the threat location. Furthermore, the participant’s demographic was also included as a set of independent variables as they can have a dramatic impact on the observed velocities and accelerations as shown in many existing databases on pedestrian dynamics^73^. The following independent variables were used:

- Distance: it represents the Euclidean distance of the aggressor
- Relative X (X) and Relative Y (Y): they represent the relative positions of the participant using an aggressor-centric, directional reference system
- Age, self-assessed exercise level, and gender (female, F).

To investigate the potential non-linearity (and asymmetric non-linearity) of the distance and the relative positions of the aggressor, we also included in the model specification the squared value of the distance (Distance^2^) and the squared and cube values of the relative positions (X^2^, Y^2^, X^3^, Y^3^). Finally, to assess the potential symmetry impact of the relative positions of the aggressor, we also considered the absolute values of these positions (Abs(X) and Abs(Y)).

Similar to the discrete analysis performed, further VR-linked predictor variables were included that were only non-zero within the VR dataset. A forward-backward stepwise variable selection procedure was used, meaning that only significant predictor variables are displayed. Therefore any non-VR-linked variable shown is significant for both datasets. However, any VR-linked variables that are present indicate a significant difference between the datasets. This is crucial to understanding the differences between paradigms.

### Participants

There were 135 participants in total across the two studies, after attrition and no-shows. Of these there were 80 participants in the PR experiment (54 male, M, and 26 female, F), and 55 participants in the VR (37M, 18F). Participants were recruited from staff and student populations with the following criteria:

- Fit and physically healthy (e.g. able to jog 100 m without stopping)
- Able to abstain from caffeine for 24 h
- Non-smoker

An exclusion criterion was used for both experimental procedures: any stress related illnesses (e.g. PTSD, high blood pressure). For the VR experiments a further exclusion criterion was used: any participants who suffer from motion sickness or conditions that could be exacerbated by VR.

Post hoc power analyses were conducted using G*Power 3.1.9.7^74^ to examine the power for the population. Within the t-tests family, a two-tailed test for the difference between two independent means (two groups) was deployed, with an alpha of 0.05, sample size group 1 being 80 (PR), sample size group 2 being 55 (VR), and a large effect size (Cohen’s d = 0.80^75^). The selection of a large effect size for the population was based on two meta-analysis studies on virtual reality exposure therapy applying behavioural and mental assessments^76,77^. The result showed a power of 0.994. Within the F-test family, a test for the linear multiple regression (fixed model, R2 deviation from zero) was deployed, with an alpha of 0.05, a total sample size being 135, the number of predictors being 28, and a large effect size (f2 = 0.35^75^). Results show a power of 0.976. Both power analyses revealed sufficient power to test the two hypotheses proposed in this study.

- Linear multiple regression (fixed model, single regression coefficient) with 28 predictors. Resulting $\alpha$: 5.14e−06, $\beta$: 1.54e−05, power 0.999.
- Means (two independent groups). Resulting $\alpha$: 0.031, $\beta$: 0.062, power: 0.937

The demographics and psychological measurements of the participants from the different experiments are detailed in Table 1. There were two statistically significant differences observed between the participants of the PR and VR experiments. The age of the PR experiments (25.0) was significantly (p < 0.001) higher than the age of the VR participants (21.3). The pre-experiment anxiety (STAI) of the PR participants (39.1) was significantly higher (p < 0.05) than that of the VR participants (36.91). No other significant differences were identified, and these differences were considered minor enough to not warrant further investigation.

There were no dropouts within the VR experiment due to simulator sickness. This number was lower than expected, and the authors believe that this was a product of the locomotion method, the training procedure, and the short duration of the VR experience. However, due to the single type of VR experiment, no formal analysis was possible.

**Table 1:** Demographic split between paradigms (mean, sd).

Value		PR (54M, 26F)	VR (37M, 18F)	Test statistic (p value)
Age		25.0 (3.88)	21.3 (2.89)	MW 647.0 (6.17e−09)***
Personality	A	2.82 (0.63)	2.91 (0.52)	$-$ 0.8915 (0.374)
	C	2.61 (0.66)	2.52 (0.62)	0.8192 (0.414)
	E	2.13 (0.76)	2.21 (0.72)	$-$ 0.5429 (0.588)
	N	2.24 (0.75)	2.42 (0.80)	$-$ 1.300 (0.196)
	O	2.63 (0.53)	2.73 (0.51)	$-$ 1.097 (0.275)
Height		173.4 (9.16)	174.6 (10.8)	0.2501 (0.803)
Weight		67.4 (12.3)	67.2 (10.3)	0.5623 (0.575)
Exercise (1–5)		3.66 (1.21)	3.65 (1.14)	MW 1676.0 (0.4977)
STAI-T		39.1 (8.33)	36.91 (7.79)	MW 1328.5 (0.035)*

*p < 0.05, **p < 0.01, ***p < 0.001, and ^†^p < 0.1.

### PR and VR comparison classification

This paper performs a quantitative comparison of movement responses between the experimental paradigms. In order to determine the differences between these paradigms, the responses are modelled using combined datasets, where the data from VR environments is also marked with a binary flag indicating its source. The comparator models use a list of predictor variables that are common to both datasets, and then a list of equivalent predictor variables that are only non-zero in the VR dataset.

The results of these models are then investigated, with any significance codes noted. Table 2 shows a colour-coded chart for assessing VR as a method of generating data on human behaviour in emergencies. If the response is the same in both VR and PR experiments, then the model results will show either no significance across both base and VR-linked variables, or only significance in the base variable (i.e. the VR-linked variables explain no more of the variance). In these instances, the colour classification is green. However, if significance is only found for the VR-linked variable, then the effect is only significant in VR environments, and the classification is orange. If significance is present in both base and VR-linked predictor variables, then the resulting classification depends on two factors: the sign and magnitude of the coefficient. If the coefficient sign is the same (i.e. both positive coefficients, or both negative), then the VR environment exaggerates the dependence on this variable, and the classification is yellow. If the sign is different between base and VR-linked variable, then further analysis is required on the standalone models investigating the individual datasets. Here there are three possible options:

1. If the significance disappears within the VR standalone model, then the effect is only present in the PR paradigm. This is classified as orange.

If significance is found within the standalone model, there are two remaining avenues:

2. The sign remains the same. In this instance the effect is present in both paradigms, but the VR environment reduces the dependence on this variable, and the consequent classification is yellow.
3. The sign is reversed. Here the effect is present in both paradigms, but with a reversed sign in VR when compared with PR environments. This is classified as red, indicating an opposing relationship between the predictor and response variables in VR and PR paradigms.

**Table 2:**
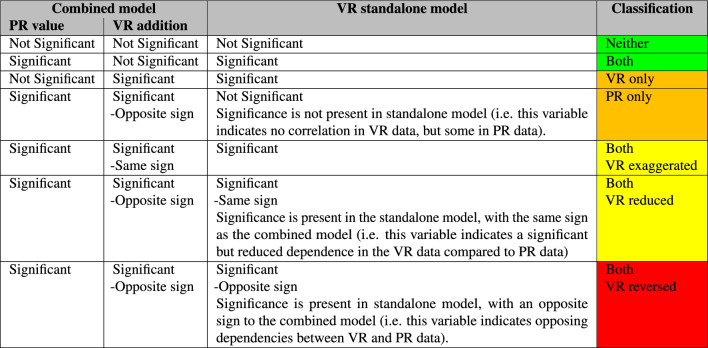
PR-VR comparison classification.

### Differences between experimental procedures

These experimental procedures were designed to be as identical as possible. As a result, the stressor and financial incentives provided to each participant were identical. Furthermore, the environmental layout was as similar as hardware and software limitations would allow (i.e. the major geometrical features were the same, but the VR environment was not photorealistic). Examples of the comparison between the environments can be seen in Figs. 1 and 2 .

Finally, the participant’s observations of other NPC responses was designed to be as similar as possible to the PR experiment. In the VR experiment, all the observed avatars movements were taken from the data obtained within the PR experiments, ensuring that the VR participant only ever observed movement responses that had actually occurred previously (i.e. rather than simulated or fake responses).

While significant efforts were made to ensure the PR and VR experimental paradigms were as similar as possible, it was inevitable that there would be some differences. The major differences are detailed here.

In the PR experiment each individual session consisted of several participants who underwent the experiment at the same time. However, given time, logistical and equipment constraints, this was not possible within the virtual experiment and therefore the VR experiment participants were not taking part in an experiment with other real participants, but rather with computer-controlled NPCs. Although measures were taken to convince the participants that the avatars they observed were real, it remains a significant difference between the experimental paradigms. As a result of this difference, the interactions between participants and observed avatars may be distinctly different from those from an experiment in which the avatars had been controlled by real participants. The post-experiment surveys for the virtual paradigm asked participants about their belief in the controlled nature of the avatars, with the majority of participants stating that they had been convinced that the avatars were real participants. This level of belief is investigated fully in “Results” section.

Owing to the automated nature of the environment, there were limitations on the available participant actions. For example, during the stressful period of the experiment, there were several activities available to the PR participants that were not available to the VR participants, including crypsis (‘playing dead’) and altruistic behaviour. There were also limitations on the possible interactions between the participants and the NPCs; these interactions were specifically limited to waving, and one instance of audio communication through a recorded message. These differences in available actions were unavoidable. However, as the VR environment was built after the PR experiment had been completed, the VR environment being tailored to investigating the effects observed in the PR experiment, specifically the impact of avatars moving on the participant’s choice to move. Therefore it is considered that the lack of these behaviours had a minimal effect on the overall results.

There was a difference between experiments in terms of the access to the surrounding areas experimental arena. The PR environment was located within a sports hall and had access to the outside world through a series of doors, whereas the VR environment had limits on where the participants could move. In both paradigms the participants were told that they could go anywhere inside or outside the building, however in the VR paradigm there was no access to an external environment.

Finally, the participants were provided different distractor tasks during the experiments. The PR experiment asked the participants to perform cognitive and physical tasks for a fixed duration of 5 min prior to the entrance of the aggressor. The virtual experiment instead asked the participants to perform an introductory session, including waving at other avatars and playing a pre-recorded introduction message. This difference was unavoidable, but was not considered significant, as both task types were simple and low intensity, and participants were aware that these tasks had no bearing on their successful completion of the experiment and their financial incentive.

### Ethical approval

 Prior to this experiment, we performed a pilot study to ensure both ethical viability and appropriate procedures^13^. All experimental protocols were approved by the Imperial College London Research Ethics Committee, all methods were carried out in accordance with relevant guidelines and regulations, and sufficient informed consent was obtained from all participants. Participants were informed of all of the potential risks (without informing of them of the true nature of the experiment and consequently reducing the ecological validity), were told they could withdraw at any point without penalty, and furthermore there were trained medical professionals present in case of unexpected adverse outcomes. Each participant was fully debriefed after the experiment and given the opportunity to reconsent to the experiment or opt out without losing their financial remuneration. As a result of these mitigation efforts, the study fully satisfied the ethical review board committee.

## Results

This section details the results obtained by the experimental procedures, specifically focusing on combining and differentiating between the two datasets. Initially this section reports the demographics of the participants, before detailing the level of self-assessed belief of the VR-participants.

This section then describes the differences in psychological responses comparing the cognitive states of the participants. Finally this section investigates the difference in movement responses of the participants, from both a discrete and continuous perspective.

### VR social belief

**Figure 3:**
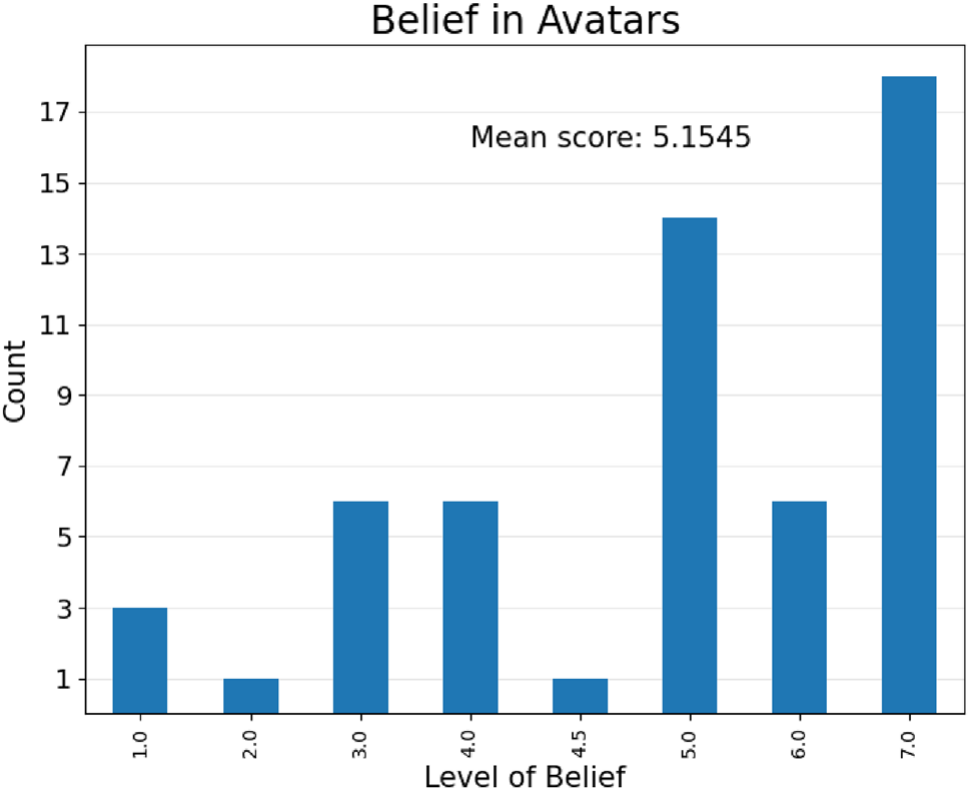
VR participant social belief levels (a value of 4.5 is allotted to the single participant who noted a response of 4–5.

One of the requirements for the participants of the VR experiment was that they believed the avatars they observed were real participants, which was encouraged using the interventions detailed in the methodology. After completing the post-experiment questionnaires, participants were informed that the NPCs were in fact all computer controlled. The participants were then asked to rate on a 7-point Likert scale the degree to which they had believed the NPCs were controlled by humans (1 = Not at all, 7 = Completely). The results obtained are shown graphically in Fig. 3. A single participant responded ‘4–5’, so an average value of 4.5 was used.

As the results indicate, the majority of the participants believed that the NPCs were human-controlled, with a mean score of 5.1545, and the most common occurrence indicating complete belief. It is unclear whether the participant responses that indicated a lack of belief were a result of a cognitive bias in desiring to appear to have understood the deception, or instead were a result of actually having understood the deception beforehand. Further to this, it is not known whether an individual’s responses differ based on this belief level. As a consequence, the VR results will now be analysed identically to the PR results, assuming the social responses of participants are based on the same degree of belief in the humanity of the avatars as if they were in a PR experiment. Future research might consider varying the level of information provided to participants prior to the experiment, to understand any changes in participant response based on their belief in the nature of the NPC.

### Psychological responses

**Figure 4:**
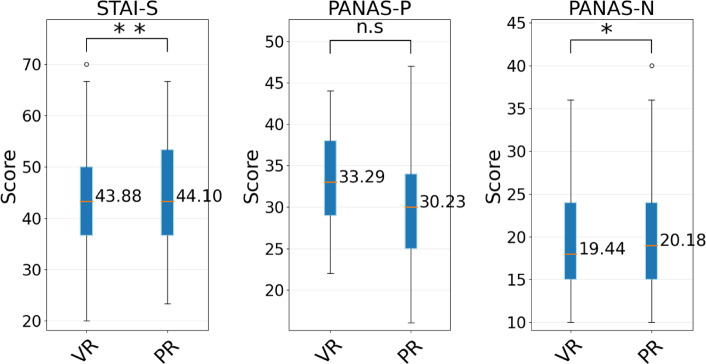
Self-reported emotional states (note: STAI-S, PANAS-P and PANAS-N were measured after the experiment).

Figure 4 graphically shows the results obtained from the psychological measures for the participants of the PR and VR experiments. Figure 4 also reports the results of the TOST, and the scores of the STAI-S and PANAS-N for VR and PR settings are equivalent. On the other hand, the TOST does not provide statistical evidence that PANAS-score are equivalent. By comparing the difference of the PANAS-P score using a traditional t-test, it is possible to identify a significant difference (p < 0.05) between the scores for the VR and PR settings. This analysis indicates that there were equivalent levels of anxiety (STAI-S) and negative emotional responses to the two settings used in this experiment (PANAS-N). Furthermore, it indicates that the participants in VR experienced a higher degree of positive emotion than those in the PR experiments.

### Discrete data

**Table 3:** Logistic model.

Combined data: move/remain still logistic regression						
Variable	Coefficient	Confidence interval	Std error	Odds-ratio	Z value	p value
PR variable						
Constant	− 3.616	[− 5.561, − 1.671]	0.993	0.027	− 3.643	0.0003***
Number reacted	0.614	[0.518, 0.711]	0.049	1.848	12.486	8.92e−36***
Number caught	0.289	[0.170, 0.409]	0.061	1.335	4.736	2.18e−06***
Distance to aggressor	− 0.018	[− 0.061, 0.025]	0.022	0.982	− 0.818	0.413
Within FOV	0.430	[− 0.021, 0.882]	0.231	1.538	1.867	0.062
F	− 0.779	[− 1.210, − 0.348]	0.220	0.459	− 3.544	0.0004***
Exercise level	0.306	[0.129, 0.482]	0.090	1.358	3.390	0.0007***
Age	− 0.020	[− 0.079, 0.038]	0.030	0.980	− 0.683	0.495
VR additive						
VR constant	− 2.447	[− 6.126, 1.232]	1.877	0.087	− 1.304	0.192
VR number reacted	0.228	[ 0.022, 0.433]	0.105	1.256	2.169	0.0301*
VR number caught	0.181	[− 0.115, 0.478]	0.151	1.199	1.199	0.230
VR distance to aggressor	0.075	[− 0.056, 0.207]	0.067	1.078	1.122	0.262
VR within FOV	− 0.771	[− 1.606, 0.064]	0.426	0.463	− 1.810	0.070
VR F	1.599	[ 0.829,2.369]	0.393	4.948	4.070	4.71e−05***
VR exercise level	− 0.350	[− 0.670,− 0.029]	0.164	0.705	− 2.136	0.0327*
VR age	0.080	[− 0.033,0.193]	0.057	1.083	1.393	0.164
Mcfadden’s pseudo $r^2$				0.415		
Log-likelihood (LLR p value)				− 459.52 (2.266e−129***)		

*p < 0.05, **p < 0.01, ***p < 0.001, and ^†^p < 0.1.

**Table 4:** Confusion matrix for combined data logistic model.

Combined data		Actual classification	
		Move	Remain still
Model classification	Move	667	87
	Remain still	126	314
Sensitivity	0.841		
Specificity	0.783		

The combined discrete movement model shows the predictor variables that are similarly relevant PR and VR paradigms, as well as those that are either only present in the PR paradigm or only in the VR paradigm.

**Table 5:**
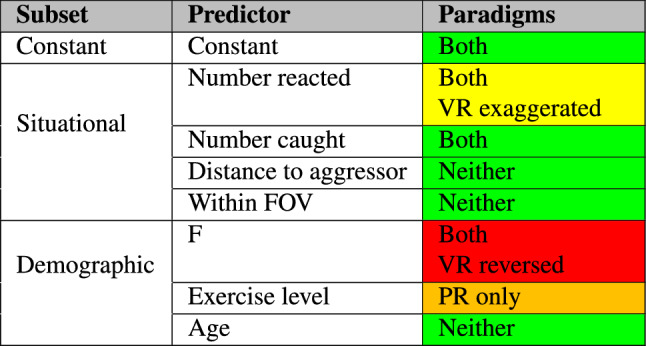
Comparison of logistic model between paradigms.

As can be seen in Table 3, the model suggests that the initial decision to move similarly unlikely in both PR and VR models, as shown by the constant parameter (coefficient = − 3.616, p < 0.001), and the lack of significance in the VR-linked variable. Additionally, the PR and VR model both indicate that a participant is more likely to move with the increasing number of other caught participants (‘Number caught’, coefficient = 0.289, p < 0.001). Both models suggest that the ‘Distance to aggressor’ variable was not significant, while the ‘Within FOV’ variable was also not significant at the 5% significance level. Finally, neither of the PR or VR models suggest significant effects based on age. However, given the limitations on recruitment (i.e. participants aged between 18 and 35), it is possible that a broader demographic might have a different outcome. This also contrasts with the results from the continuous analysis, detailed below. The confusion matrix for this model is provided in Table 4.

In contrast, both models the observed number of participants that decided to move, (“Number reacted”), yielded a positive relationship with the decision to move (coefficient = 0.614, p < 0.001). However, this effect was significantly stronger in the VR dataset (additional coefficient = 0.2276, p = 0.030). Additionally, in the PR experiment, participants who had self-assessed with higher exercise levels were also significantly more likely to move (coefficient = 0.306, p < 0.001), whereas this effect was not seen in the VR environment (additional coefficient = − 0.350, p < 0.05). Finally, in the PR dataset, female participants were less likely than male participants to decide to move (coefficient = − 0.7791, p < 0.001), whereas in the VR dataset this effect was reversed, with female participants more likely to decide to move than male participants (additional coefficient = 1.599, p < 0.001). This is the only significant reversed response detected between paradigms. These results are shown in a colour-coded format in Table 5, using the coding taxonomy defined in Table 2.

### Continuous data

This section will show the results of the multivariable linear regression, identifying common features and unique elements between the two paradigms. The final part of this analysis will provide a colour-coded table that assesses the overlap in continuous movement responses between paradigms.

Table 6 shows that there is a positive constant value within the acceleration models that is present in both datasets, with the additional positive VR constant implying people accelerate more within VR. Self-assessed exercise level has a positive relationship with acceleration, while age has a negative relationship with acceleration, across both VR and PR paradigms. There are several differences in the dependence of acceleration on position-based predictor variables. Specifically, the model suggests some unique predictor variables in VR, including in *X*, Abs(X), *Y*, Distance and Distance^2^. Female participants are seen to accelerate more than male participants in PR experiments, and less than male participants in VR experiments.

There is a significantly higher positive constant for velocity within VR environment, while both paradigms show a negative relationship with age, and a positive relationship with self-assessed exercise. There are multiple predictor variables that are present solely in the VR model, including Distance, Distance^2^, *X*, $X^2$ $X^3$, Abs(X), $Y^3$, and gender. There is also a reversed dependency on *Y*.

Both paradigms show that tangential acceleration has a positive constant component, while this is exaggerated in the VR experiments. Both paradigms also show a significant relationship with $X^2$ and $Y^3$, as well as with a relationship with Distance, which is exaggerated in the VR experiments. Tangential acceleration is shown to be reduced with age and increased with self-assessed exercise level. The VR model shows a unique dependence on Distance^2^, *X*, Abs(X) and *Y*. Finally, the VR model shows a reversal of the dependence on gender, where female participants accelerate more in PR experiments and male participants accelerate more in VR experiments.

Both datasets produce a positive constant term within the radial acceleration model, although this is significantly exaggerated within the VR model. The model suggests that there is a VR specific negative relationship between radial acceleration and gender, as well as *X*, Abs(X) and *Y*.

Finally, the model for radial velocity shows that both paradigms see a positive constant term within the regression, which is again significantly higher in the VR model. Both models also see a negative relationship with $Y^3$, Abs(Y) and age. The VR model suggests a unique relationship between radial velocity and numerous predictors, including Distance, Distance^2^, *X*, $X^3$, Abs(X), *Y*, self-assessed exercise level, and gender.

**Table 6:**
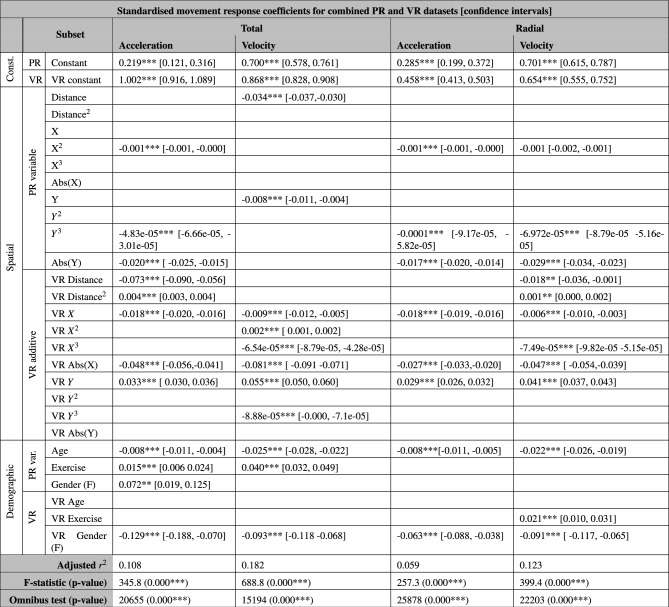
Combined standardised models.

*p < 0.05, **p < 0.01, ***p < 0.001, and ^†^p < 0.1.

Table 7 combines all of the models into a single colour-coded chart, showing the level of overlap between the paradigms. It uses the same colour-coding system as detailed in Table 2. This is a simple method of summarising the quantitative information shown in Table 6, by movement type and by predictor variable subset. When comparing the spatial elements within this table, it can be seen that there are significant overlaps between the VR and PR experimental paradigms, while there is only a singular reversed behavioural response within the X variable. The demographic predictor subset shows overlap in both age and exercise variables, while there are differences in gender-specific responses across all responses. It can be concluded from this table that there are significant overlaps in the data observed, and that the VR environments utilised in these experiments are a reasonable approximation of real-world continuous movement. However, it is also concluded that there is a significant difference in response type based on gender between the experimental paradigms, and all future work should be aware of this difference when inferring any relationships. The numerous “VR only” boxes within the spatial subset are considered to be a product of the lack of noise in the VR measurements.

**Table 7:**
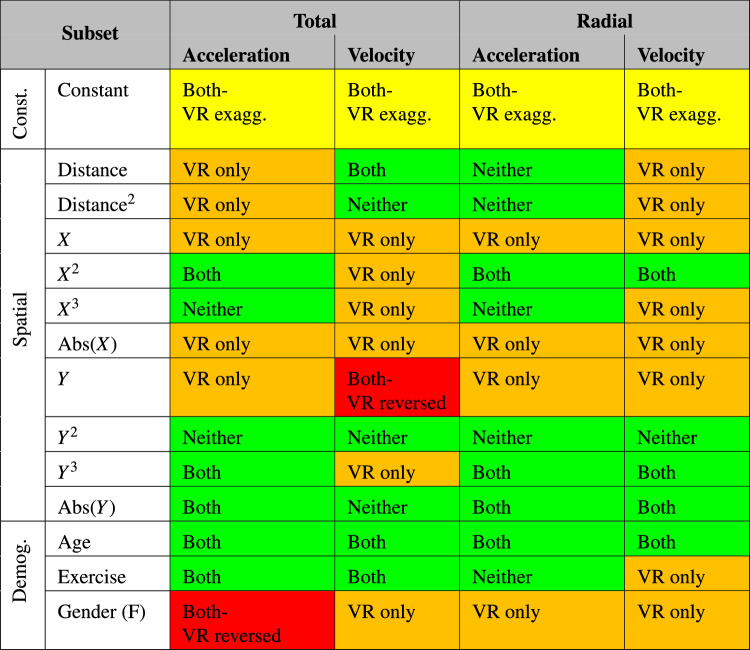
Similarities between movement types within PR and VR datasets.

## Discussion

There is little doubt that a variety of emergency situations would greatly benefit from an informed model of human movement responses, however, any attempt to develop such a model suffers from a lack of appropriate data. Currently, experimental approaches to data gathering in such scenarios focus upon physical reality (PR)-based experiments (such as drills), and virtual reality (VR)-based experiments (ranging from desktop-surveys to fully immersive environments), each of which has its accompanying drawbacks. This paper explored whether the similarities between the results of an emergency scenario, involving a knife-wielding aggressor and 135 participants, showed sufficient promise for VR to be a genuinely realistic data gathering approach when compared to PR. To avoid any personality bias, we verified if there was any difference in terms of personality between the participants of the VR and PR experiments. The results in Table 1 for the “Big 5” show no statistical difference between the two groups. We instead measure the emotional state of the participants to assess the impact of the two experiments on the negative emotions of the participants.

The results in Fig. 4 show statistical equivalence in terms of negative emotions (see PANAS-N) and level of anxiety (see STAI-S) between the two groups after the experiments. On the other hand, we observed that the VR experiment generated more positive emotions than the PR experiment. This is explained by the fact that VR experiments are still a slight novelty, leading to slightly elevated positive emotions. The results of this study revealed several similarities between the results obtained in PR and VR experiments. Initially, the psychological responses between participants were almost identical, with the only statistically significant difference arising in the measure of positive emotion. This is explained by the fact that VR experiments are still a slight novelty, leading to slightly elevated positive emotions.

When considering movement, this study split participant responses into two different categories: discrete and continuous. Table 5 shows the large overlap between paradigms when considering discrete responses, where no statistically significant differences were detected between the paradigms when considering several factors: the initial likelihood to move, age, the number of other participants caught, the distance to the aggressor, and the direction of aggressor motion.

In contrast to these similarities, the combined model also shows an exaggerated social dependence within VR (‘Number reacted’), and a unique dependence on self-assessed exercise level within the PR environment. This dependence on exercise level in the physical experiments is suggested to be partially explained by the armswinging locomotion method within VR, which did not require physical exertion. A major difference is seen in the responses of different gender participants, where female participants are less likely to move within a physical environment, but more likely to move within a virtual environment. It is suggested that this reduced likelihood of moving in a physical environment could be a learned effect, but this would not explain their increased likelihood of moving in the virtual environments. Therefore this difference in the responses of different participant genders across paradigms is unexplained, and could represent a fruitful area of sociological research. Overall, the majority of the predictor variables were statistically indistinguishable, suggesting a strong similarity in the responses between paradigms.

This methodology also revealed that the continuous datasets had many significant similarities between paradigms. In fact, 27/56 ($\sim$ 48%) of the points of comparison the predictor variables were statistically indistinguishable, and a further 4 ($\sim$ 7%) had the same valence in both paradigms, while the response in one of the paradigms was significantly exaggerated. Of the remaining 25 points of comparison, 23 predictors were statistically significant in the VR environment only, which is suggested to be at least partially a result of the lower noise within VR environments, as well as potentially a product of the locomotion method utilised. Finally, there was a significantly reversed response between the paradigms in within the gender predictor variable for total acceleration, as well as within the y-component for total velocity. This gendered difference in responses is considered unexplained, and should be considered in all future VR experiments. The y-component difference is also unexplained, however, could be related to depth perception within virtual environments.

While this study aimed to ensure as similar conditions as possible between the paradigms, the comparison between participant demographic showed two points of difference: age and pre-experiment cognitive state. The difference in age is suggested to be a result of two major factors. Primarily it is thought that the relative attractiveness of VR as an experimental paradigm to younger populations may have reduced the average age of participants. Additionally, the experimental timings may have led to the difference, as the PR experiment was performed in December, prior to university exams, when undergraduate participants may have been revising. The difference in pre-experiment cognitive state is suggested to be at least partially a result of the fact that the PR experiment was performed as a group, while the VR experiment was performed individually. These are both considered small differences, and therefore the results from participants for both experiments were treated identically.

It is considered that there exists a requirement for sufficient complexity in a VR environment for the participants to be able to produce similar responses as they would in PR environments. This is required for participants to act ‘naturally’, as though they were in a real environment. For example, this study investigated participant movement in PR and VR environments after participants had been provided identical movement options (i.e. continuous movement with variable movement speed in any direction). It is suggested that there would have been different responses if participants in the VR environment had been provided limited responses options (e.g. teleporting to specific locations only), or forced responses (e.g. a prompt saying ‘Do you want to move?’). A further consideration is that there are limitations to the types of data obtained within VR experiment, as any complex behaviours need to be implemented before they can be performed by a participant. As an example, participants in physical experiments can choose to hide, or perform crypsis, but it is much more difficult to allow participants in VR environments to perform the same actions.

This paper highlighted two hypotheses based upon the literature review and subsequently examined the responses of participants in two almost identical experiments, across two different paradigms. This methodological solution is in line with some of the existing attempts to validate the VR paradigms (see, for instance, Kinateder and Warren^49^). This solution can provide a more accurate approach for VR validation instead of comparing VR data with historical data of previous disasters (see, for instance, Arias et al.^51^ and Arias et al.^48^). It did so while obtaining relevant psychological, demographic and spatial data, overcoming the limitations identified in previous studies in the “Literature review”. Finally it modelled the resulting movement in a way that can be easily applied to pedestrian dynamics models. Overall, it is seen that there is a large degree in overlap between the responses between VR and PR experimental paradigms within the factors affecting these responses. As such, the results support our first hypothesis (i.e., an overall agreement between the VR and PR data from the hostile emergency). The previous literature had identified the requirement for validation^21^, as well as some initial indications regarding the validity of VR data^51^. We argue that this study has gone further than previous attempts, and provides quantitative evidence that participants react similarly in VR and PR, as well as providing a quantification of any deviation (e.g. through gender disparities). These results satisfy the second hypothesis (i.e., the intensity of how factors affect the participants’ response might differ depending on the paradigm), while also providing some much needed detail on the direction of fruitful future research. Improving upon this analysis will require several further elements, including more data, more predictor variables such as neighbour participant states (e.g. speed, acceleration, and direction), or a more sophisticated modelling approach, incorporating time-series dependence.

## Conclusion

This study analysed the results taken from an experiment performed in two separate paradigms, observing any differences. There were minimal differences observed in the psychological responses to the study, and a large degree of similarity in participant movement responses, both in the discrete choices and continuous movement.

This study therefore concludes that VR can be used to obtain discrete movement choices that will accurately mimic data from PR experiments. However, when obtaining this data, the experimental team should be aware of potential confounding effects from increased social dependence and from gendered effects. Additionally, this study concludes that VR can be used to obtain continuous movement data which will mimic data obtained from PR experiments.

There is a limitation in this analysis in that there is still a question surrounding the utility of these datasets when considering real-world responses. This may be intractable, given the inherent difficulties in obtaining controlled data surrounding how people move when they are in moments of extreme danger. However, given these limitations, we believe that this study has made significant strides in improving our understanding of human behaviour in emergencies, and how we investigate these scenarios.

Further work in this area should consider developing the experimental design of the VR environment, for example performing experiments with groups of participants who are co-located in physical and digital environments. This will be a significant improvement to the experimental design, as it reduces experimental deception, and increases the parallels with the PR experimental paradigms. Additionally, a single locomotion technique (armswinging) was used, therefore future research should investigate the validity of different locomotion techniques, including omnidirectional treadmills and free movement.

This conclusion significantly adds to the evidential basis for using VR environments as a data generating paradigm, especially for emergency scenarios. This is crucial, as the VR paradigm allows for far more stressful and realistic environments to be portrayed (e.g. a marauding terrorist firearm attack, MTFA), which would otherwise be very complex to perform in a PR environment. VR represents a paradigm with drastically reduced ethical and health and safety concerns, as well as improved logistical requirements, while maintaining the ability to perform individual and group experiments. This ability has a number of major implications: (1) it will allow for the improved design of infrastructure against hostile attacks, and the case can be made that prior to the approval of the promising designs, VR exercises be conducted as outlined above (2) with existing critical infrastructure, e.g. major transport terminals, VR exercises can be used by the relevant government agencies and security services to determine the best guidance for both first responders as well as for the general public. By understanding the emotional and movement responses to these hostile attacks, and consequently any guidance and management policies that are implemented to control these events, lives will ultimately be saved and physical harm reduced.

## Data Availability

The datasets generated during and/or analysed during the current study are available from the corresponding author on reasonable request.
